# The Ayushman Bharat Pradhan Mantri Jan Arogya Yojana and the path to universal health coverage in India: Overcoming the challenges of stewardship and governance

**DOI:** 10.1371/journal.pmed.1002759

**Published:** 2019-03-07

**Authors:** Blake J. Angell, Shankar Prinja, Anadi Gupt, Vivekanand Jha, Stephen Jan

**Affiliations:** 1 The George Institute for Global Health, University of New South Wales, Sydney, Australia; 2 School of Public Health, Post Graduate Institute of Medical Education and Research, Chandigarh, India; 3 National Health Mission, Government of Himachal Pradesh, Shimla, Himachal Pradesh, India; 4 The George Institute for Global Health, University of New South Wales, New Delhi, India; 5 University of Oxford, Oxford, United Kingdom

## Abstract

In an Essay, Blake Angell and colleagues discuss ambitious reforms planned to expand coverage of the health system in India.

**Summary points**

- Public spending on healthcare in India is amongst the lowest in the world at just over 1% of gross domestic product (GDP), and the Indian health system is characterised by substantial shortcomings relating to workforce, infrastructure, and the quality and availability of services.
- The Ayushman Bharat Pradhan Mantri Jan Arogya Yojana (AB-PMJAY), approved by the Indian government in March 2018, is an ambitious reform to the Indian health system that seeks to provide financial health protection for 500 million of the most vulnerable Indians and halt the slide of the 50–60 million Indians who fall into poverty annually as a result of medical-related expenditure.
- There is a need for wide reforms across public and private providers of care if India is to meet its stated aims of providing universal health coverage (UHC) for its population. The success of the program will rely on a reformed and adequately resourced public sector to lead implementation, delivery, and monitoring of the scheme.
- While there are significant challenges facing the program, by providing the impetus for system-wide reform, AB-PMJAY presents the nation with a chance to tackle long-term and embedded shortcomings in governance, quality control, and stewardship and to accelerate India’s progress towards the stated goal of UHC provision.
- Implementation and ongoing operation of the program need to be carefully monitored to ensure that it is meeting its aims in a sustainable manner and that negative unintended consequences are avoided.

## Introduction

Successive Indian national governments have stated a commitment to achieving universal health coverage (UHC). In spite of this, UHC remains an elusive aim, and the Indian health system continues to be characterised by substantial shortcomings relating to workforce, infrastructure, and the quality and availability of services. Public expenditure on healthcare in India remains at levels amongst the lowest in the world. The government of India approved the Ayushman Bharat Pradhan Mantri Jan Arogya Yojana (AB-PMJAY) in March 2018 and has hailed the program as a historic step towards achieving UHC in India. The scheme aims to publicly fund the healthcare of up to 500 million people and, if it lives up to its potential, represents a unique opportunity to institutionalise quality healthcare free at the point of service for the most marginalised Indians, improving the health of the population and drastically reducing or eliminating medical-related impoverishment. While many have already questioned the likelihood of successful implementation of the AB-PMJAY, the vast ambition of the program presents an opportunity to pursue the systemic reform that India requires to meet its UHC aims. This will require an injection of resources into a chronically underfunded health system, but this must be accompanied by a focus on the interrelated issues of governance, quality control, and stewardship if the scheme is to sustainably accelerate India towards UHC.

## The policy context

The Indian health system comprises a complex mix of various levels of government decision makers and providers, private companies, and other nongovernment service providers. The country has a chronic shortage of doctors and other healthcare providers, who tend to be concentrated in the urban centres, leaving large parts of the country underserved [1,2]. Notwithstanding increases in real terms over recent decades, government expenditure on health in India ranks amongst the lowest in the world at a little over 1% of GDP [3]. Consequently, the system depends heavily on out-of-pocket payments charged to patients at the point of care. Such payments limit access to care and have a disproportionate economic impact on the poor [4]. Impoverishment in India as a result of healthcare costs is common for patients and their families, with an estimated 50–60 million people pushed into poverty each year as a result of medical-related expenditure [5].

A raft of policies have been implemented by state and national governments over recent decades to improve healthcare coverage in India [2]. Most notably, the National Rural Health Mission was established in 2005 [6] by the central government to provide universal access to care for rural residents, which was then joined by the National Urban Health Mission to create the National Health Mission in 2014 [7]. These policy initiatives were accompanied by an increase in health system infrastructure such as community and primary healthcare centres [2]. Along with a number of state and national schemes such as the Rashtriya Swasthya Bima Yojana launched in 2007 that covered hospital expenses of up to INR 30,000 (approximately US$420) for families living below the poverty line, it was estimated that, by 2010, up to 25% of India’s population had some level of financial protection for healthcare costs [8]. While these and similar schemes have been accompanied by ambitious mandates, in many cases their impact on financial risk protection has been limited by insufficient resourcing and coverage gaps [2,8–13].

## Modicare and UHC

In this context, the cabinet of the Indian government approved the ambitious AB-PMJAY in March, 2018. The scheme, colloquially referred to as “Modicare” after Indian Prime Minister Narendra Modi, aims to build on existing schemes to provide publicly funded health insurance cover of up to 500,000 Indian rupees (over US$7,000) per family per year to about 100 million families (500 million people, 40% of India’s population) [14,15]. The scheme builds on the previous programs outlined above (for example, the National Health Mission still forms the basis of primary care under the new program [16]) and has been designed to be implemented to either take over or operate alongside state-based programs, but has a broader remit in terms of the services covered and the amount of coverage that each individual is entitled to. The government has so far allocated 100 billion rupees (almost US$1.5 billion) to the program for 2018–2019 and 2019–2020 [14]. Currently, the country spends about US$64 per person on healthcare, two-thirds of which is privately financed by user fees [17]. As such, current UHC initiatives in India centred on AB-PMJAY alongside state-based programs such as those in Andhra Pradesh, Telangana, Tamil Nadu, Karnataka, and Kerala represent, as a whole, one of the most ambitious ever health and, one could argue, poverty-alleviation programs ever launched.

Details of AB-PMJAY initially emerged in a piecemeal way via government press releases and media interviews [18–20]. More recently, government guidelines for implementing different parts of the scheme have been released [15,16]. Eligibility for the scheme is determined based on deprivation criteria measured in the 2011 Socio-Economic Caste Census. There is no limit to the number of family members covered, and benefits will eventually be India-wide (if all states and union territories sign up to the program). This means that a beneficiary will be allowed to take cashless benefits from any public or empanelled private hospital across the country. State health authorities will lead the implementation of the AB-PMJAY, and states are free to continue to provide existing programs alongside the national program or integrate them with the new scheme. States will also be able to choose their own operating model to either use the expenditure to pay a private insurance provider to cover services, provide services directly (as elected by Chandigarh and Andhra Pradesh, for example), or a mix of the two (as in Gujarat and Tamil Nadu) [20]. Expenditure under the program will also be shared between the central and state governments in a prespecified ratio depending on the legislative arrangements and relative wealth of the states, with the Indian government covering between 60%–100% of expenditure [18,20]. A pilot of the program, involving only public hospitals, was launched in August 2018 across 110 districts in 14 states and union territories, with a large number of private hospitals having since been empanelled under the program [21].

## The challenges of governance and stewardship

UHC aims to ensure access to quality essential healthcare services and medicines for populations without exposing them to the risk of financial hardship [22]. Progress towards UHC must be seen in light of the severe challenges facing the Indian system. The country is beset by deficiencies in the resources available to fund healthcare, the skilled workforce and infrastructure available to provide care, and oversight of healthcare provision. Private providers have become the dominant provider of care in India, and thus UHC is unlikely to be achieved without engagement with this sector [2,23,24]. The profit motive that drives the behaviour of these providers, however, has led to concerns that services may be encouraged to sometimes act against the public interest. Regulation and oversight of these providers in low- and middle-income nations is often poor. There is evidence from across low- and middle-income countries that private providers more frequently deviate from evidence-based practice, have poorer patient outcomes, and are more likely to provide unnecessary testing and treatment [25], and the data that do exist from India have mirrored these findings [26]. At the same time, public providers in India have been shown to face significant governance challenges as well, with services shown to be rife with absenteeism, of poor quality, and nonexistent in many areas of care. Corruption at all levels of the system from doctor training to investment decisions remains an issue [2].

Policy interventions to progress India towards UHC need to factor in these difficulties and make tangible inroads into overcoming them. Institutional inefficiencies, common in health systems across the world, are often difficult to change once embedded because change often creates winners and losers. The size and scope of the announced program, however, presents something of an opportunity to overcome some of this fragmentation and set India onto an optimal path to UHC if it is able to constructively work to overcome these challenges. Fundamental to doing so will be ensuring appropriate governance and quality of the healthcare provided to the population. Few details have emerged as to how the interrelated issues of governance, monitoring, and accountability will be managed under the scheme to progress India towards successful implementation of AB-PMJAY and ultimately UHC. As new services are provided and coverage increased, successful implementation will require a parallel concerted push towards quality assurance, appropriate governance, and appropriate referral pathways in both public and private healthcare providers. Given the importance of private providers in India, there is a need to strengthen the stewardship function of the government to monitor the provision of care from these providers. This could occur in a number of ways, such as through the development of robust referral pathways for patients, quality audits of providers, incentives to improve the efficiency and quality of care, strategic purchasing, and a general strengthening of the capacity of the public sector to effectively contract with and regulate the private sector.

## Conclusion

The AB-PMJAY offers a unique opportunity to improve the health of hundreds of millions of Indians and eliminate a major source of poverty afflicting the nation. There are, however, substantial challenges that need to be overcome to enable these benefits to be realised by the Indian population and ensure that the scheme makes a sustainable contribution to the progress of India towards UHC. UHC has become a key guiding target for health systems around the world under the Sustainable Development Goals to improve the health of the global population and overcome the scourge of medical-related impoverishment. The success of UHC is measured by the access of health services across the population, the types of services that are available, and the financial protection offered to the population. While there are obvious resource constraints in implementing AB-PMJAY, the success—or otherwise—of the scheme in making progress across these three measures will also depend on overcoming a number of existing and interrelated structural deficiencies of the Indian system such as issues of public and private sector governance, stewardship, quality control, and health system organisation. To do so will require careful monitoring of the implementation of the program to track progress against key budgetary, service, and financial-protection measures and guard against unintended consequences. In many cases, current arrangements in these areas can be seen to be a product of vested interests and a system that is not designed to reward positive change. Altering these incentives to promote universal and quality care for all Indians will require widespread reform, intervention, and leadership across all levels of the Indian system. Thus, whilst these weaknesses pose a threat to the ability of proposed reforms to meet their ambitious objectives, by providing the impetus for systemic reform, AB-PMJAY presents the nation with a chance to tackle long-term and embedded shortcomings in governance, quality control, and stewardship.

## Abbreviations

AB-PMJAY: Ayushman Bharat Pradhan Mantri Jan Arogya Yojana
GDP: gross domestic product
UHC: universal health coverage
